# “We survived the pandemic together”: The impact of the COVID-19 pandemic on Canadian families living with chronic pain

**DOI:** 10.1080/24740527.2022.2157251

**Published:** 2023-02-06

**Authors:** Tieghan Killackey, Sabine Soltani, Melanie Noel, Kathryn A. Birnie, Manon Choinière, M. Gabrielle Pagé, Lise Dassieu, Anaïs Lacasse, Chitra Lalloo, Patricia Poulin, Samina Ali, Krista Baerg, Marco Battaglia, Fiona Campbell, Vina Mohabir, Fareha Nishat, Rachel Kelly, Tatiana Lund, Ariane Isaac-Bertrand, Myles Benayon, Isabel Jordan, Jennifer Stinson

**Affiliations:** a Child Health Evaluative Sciences, The Hospital for Sick Children, Toronto, Ontario, Canada; b Department of Psychology, University of Calgary, Calgary, Alberta, Canada; c Department of Psychology, Alberta Children’s Hospital, Hotchkiss Brain Institute, Calgary, Alberta, Canada; d Department of Anesthesiology, Perioperative, and Pain Medicine, University of Calgary, Calgary, Alberta, Canada; e Department of Anesthesiology and Pain Medicine, Faculty of Medicine, Université de Montréal, Montreal, Quebec, Canada; f Research Center of the Centre hospitalier de l’Université de Montréal, Montreal, Quebec, Canada; g Department of Psychology, Université de Montréa, Montreal, Quebec, Canada; h Department of Biomedical Sciences, Université de Montréal, Montreal, Quebec, Canada; i Department of Health Sciences, Université du Québec en Abitibi-Témiscamingue, Rouyn-Noranda, Quebec, Canada; j Institute of Health Policy, Management & Evaluation, University of Toronto, Toronto, Ontario, Canada; k Department of Anesthesiology & Pain Medicine, Faculty of Medicine, University of Ottawa, Ottawa, Ontario, Canada; l Department of Psychology, The Ottawa Hospital, Ottawa, Ontario, Canada; m Clinical Epidemiology Program, The Ottawa Hospital Research Institute, Ottawa, Ontario, Canada; n Departments of Pediatrics, Faculty of Medicine & Dentistry, University of Alberta, Edmonton, Alberta, Canada; o Department of Emergency Medicine, Faculty of Medicine & Dentistry, University of Alberta, Edmonton, Alberta, Canada; p Women and Children’s Health Research Institute, University of Alberta, Edmonton, Alberta, Canada; q Department of Pediatrics, University of Saskatchewan, Saskatoon, Saskatchewan, Canada; r Department of Pediatrics, Saskatchewan Health Authority, Saskatoon, Saskatchewan, Canada; s Department of Psychiatry, University of Toronto, Toronto, Ontario, Canada; t Centre for Addiction and Mental Health CYEA programme, Toronto, Ontario, Canada; u Department of Anesthesia and Pain Medicine, Hospital for Sick Children, University of Toronto, Toronto, Ontario, Canada; v Department of Anesthesia and Pain Medicine, University of Toronto, Toronto, Ontario, Canada; w Summer Research Student, Child Health Evaluative Sciences, the Hospital for Sick Children, Toronto, Ontario, Canada; x Internal Medicine Residency Program, McMaster University, Hamilton, Ontario, Canada; y Parent partner, British Columbia, Canada; z Lawrence S. Bloomberg Faculty of Nursing, University of Toronto, Toronto, Ontario, Canada

**Keywords:** COVID-19, chronic pain, qualitative research, pediatric pain

## Abstract

**Introduction:**

Pediatric chronic pain is a significant problem in Canada, affecting one in five youth. This study describes the impact of the pandemic on the experiences of Canadian families living with chronic pain through interviews with youth living with chronic pain, parents, and siblings.

**Methods:**

Employing a qualitative descriptive design, in-depth semistructured interviews were completed with Canadian youth living with pain, as well as parents and siblings. Participants were not required to be related. Interviews were analyzed using a reflexive thematic analysis approach.

**Results:**

Forty-four interviews were completed with 14 parents, 19 youth with chronic pain, and 11 siblings from across the country. Three key themes were developed: (1) absorbing and shifting: the toll of the pandemic on the family system (e.g., loss of coping mechanisms, shifting roles to respond to the pandemic), (2) social ambiguity and abandonment (e.g., social sacrifice and abandonment by the health care system), and (3) building community resilience: familial adaptation to the pandemic (e.g., family cohesion, confidence, and self-management).

**Discussion/Conclusions:**

Youth, parents, and siblings reported that the pandemic impacted coping strategies across the family system. These results outline the challenges youth experienced managing their pain and overall health throughout the pandemic and the resilience built within families during this time. Going forward, it would be relevant to examine how racialized and structurally marginalized youth with chronic pain and their families experienced the pandemic. Future research should examine how unexpected benefits of the pandemic (e.g., increased confidence and self-management) may be sustained into the future.

## RÉSUMÉ

**Introduction**: La douleur chronique pédiatrique est un problème important au Canada, qui touche un jeune sur cinq. Cette étude décrit les répercussions de la pandémie sur les expériences des familles canadiennes vivant avec la douleur chronique par des entrevues avec des jeunes vivant avec une douleur chronique, des parents et des frères et sœurs.**Méthodes**: À l’aide d’un devis descriptif qualitatif, des entretiens semi-structurés approfondis ont été réalisés auprès de jeunes Canadiens vivant avec la douleur, leurs parents, et leurs frères et sœurs. Il n’était pas nécessaire que les participants aient un lien familial. Les entretiens ont été analysés à l'aide d'une approche d'analyse thématique réflexive.**Résultats**: Quarante-quatre entretiens ont été réalisés auprès de 14 parents, 19 jeunes souffrant de douleur chronique et 11 frères et sœurs de tout le pays. Trois thèmes clés ont été abordés : (1) l'absorption et le changement : le coût de la pandémie pour le système familial (p. ex., perte de mécanismes d'adaptation, changement de rôles pour réagir à la pandémie), (2) l'ambiguïté sociale et l'abandon (p. ex., le sacrifice social et l'abandon par le système de soins de santé), et (3) le renforcement de la résilience communautaire : adaptation familiale à la pandémie (p. ex., cohésion familiale, confiance et auto-prise en charge).**Discussion/Conclusions**: Les jeunes, les parents et les frères et sœurs ont signalé que la pandémie avait eu des répercussions sur les stratégies d'adaptation dans l'ensemble du système familial. Ces résultats décrivent les défis rencontrés par les jeunes pour prendre en charge leur douleur et leur santé globale tout au long de la pandémie, ainsi que la résilience démontrée par les familles pendant cette période. À l'avenir, il serait pertinent d'examiner comment les personnes racialisées et les jeunes structurellement marginalisés souffrant de douleur chronique et leurs familles ont vécu la pandémie.Les recherches futures devraient examiner comment les avantages inattendus de la pandémie (par exemple, une confiance accrue et l'auto-prise en charge) peuvent être maintenus à l'avenir.

## Introduction

There is growing professional awareness about chronic pain in children and adolescents.^1,2^ In Canada, the prevalence is conservatively estimated at 20% of the population, or >1 million Canadian youth reporting pain lasting longer than 3 months.^3–5^ Alongside challenging physiological symptoms, studies have demonstrated that pediatric chronic pain is associated with an increased risk of harmful psychosocial impacts on the mental health and functioning of youth and families.^6,7^ Children and adolescents with chronic pain may have higher risk of experiencing psychological distress, including feelings of anxiety, depression, and posttraumatic stress disorder.^8–10^ Experiences of insomnia,^11^ substance use,^12,13^ and a lower quality of life^14^ have also been reported among youth living with chronic pain, which highlights the increased risk of additional challenges this population may face as they transition into adulthood.

Since March 2020, there has been a significant shift in the lives of those with chronic pain brought on by the global COVID-19 pandemic, where public health–related restrictions transformed personal, social, economic, workplace, and health care environments.^15–18^ Recent literature has suggested that COVID-19 may have a greater impact on people with chronic pain when compared to others; some studies have documented improvement of chronic pain symptoms due to increased flexibility and lifestyle changes.^19–22^ Those with chronic pain have been shown to experience a heightened awareness of physical symptoms, as well as the worsening of their pain attributed to psychosocial stressors, sleep interference, and a lack of physical activity.^20^ Despite the onset of both new symptoms as well as exacerbations of preexisting pain, many youth and families experienced reduced access to both medical and mental health care during the pandemic.^23,24^ These circumstances contributed to patients reporting increased anxiety, fear, and helplessness and, in some cases, resulted in delayed diagnoses and higher complication rates.^25^ Prior to the COVID-19 pandemic, the role of family had been shown to be especially influential on pediatric pain experiences as well as quality of life.^26–29^ However, COVID-19-related stressors have shown a link between the level of parent difficulty and hardships with their child’s emotional distress.^30^ Early longitudinal studies with youth with chronic pain and their families suggest that pain interference and well-being have improved when compared to before the pandemic.^21,29^ However, youth and their families who experienced greater personal impact (e.g., economic stress, job instability, housing insecurity) during the pandemic have been shown to develop worsened anxiety,^21^ pain, and parent insomnia.^21,29^ Overall, research has varied widely regarding the impact of the pandemic on youth living with chronic pain; however, few studies have examined this phenomenon at the family system level with both caregivers and siblings of youth living with pain. There are relatively few studies that have examined the experience of siblings of young people with chronic pain, and the majority of research on siblings that does exist has used quantitative methods to explore the biological or physical components of pain.^31^ Qualitative research can offer additional insights on the experiences of siblings of youth with chronic pain.^32^ This is particularly relevant when examining the roles and responsibilities of siblings and the changes in family dynamics that may have occurred as a result of the COVID-19 pandemic.^31,33,34^ The goals of this study were to explore the experience of living through the COVID-19 pandemic for youth with chronic pain, as well as siblings and parents of youth with chronic pain across Canada, and describe this experience using the theoretical foundation of the family adjustment and adaptation response model^35^ to develop a substantive account.

## Methodology

### Participants and Design

**Figure 1.**
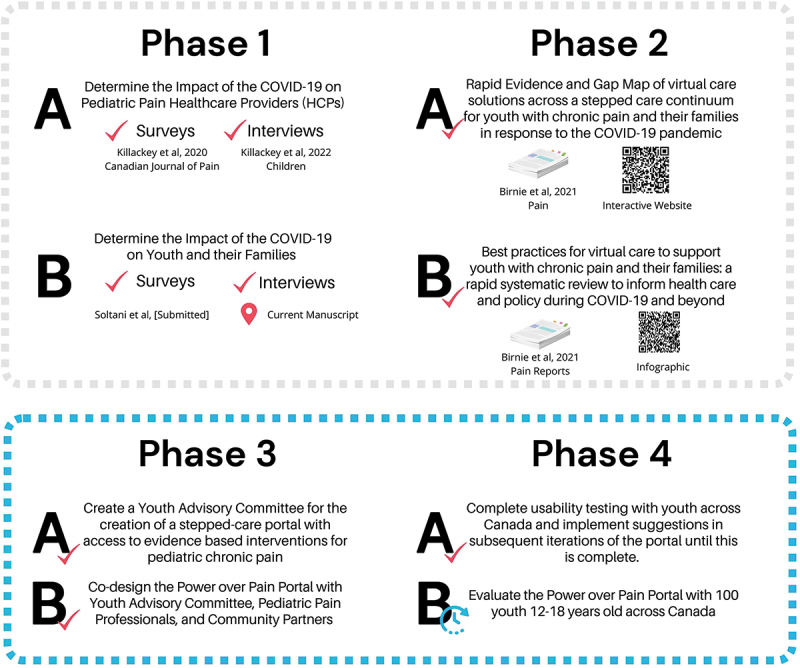
Summary of completed and future phases of the development of a stepped-care intervention for pediatric chronic pain.

This study was guided by Sandelowski’s^36^ approach to descriptive qualitative design, with the goal of describing the experiences of youth living with chronic pain and siblings and parents of youth living with pain during the COVID-19 pandemic. Eligible participants were identified from a list of candidates who had opted in for an interview following the completion of a national online survey as part of a wider program of research. The national online survey was implemented to explore the effects of the pandemic on pain and mental health experiences of youth with chronic pain, as well as their siblings and caregivers.^37^ This survey was disseminated widely to children and adolescents aged 8 to 18 years and living with pain, parents/caregivers of youth with chronic pain, and siblings of youth with chronic pain. Every pediatric multidisciplinary pain clinic in Canada sent the survey to their current patients and families; surveys were also sent through national organizations such as the Canadian Pain Network, Solutions for Kids in Pain, and CHILD-BRIGHT and shared widely on social media platforms including Twitter, Instagram, and TikTok. The schema for the larger research program is presented in Figure 1.

Purposive sampling was used, and participants were recruited with a focus on maximum variation in age, gender, geographical location, ethnicity, and socioeconomic status. Eligible youth participants were required to be between the ages of 8 and 18 years and be able to speak and read English. Siblings were required to be between the ages of 8 and 25 years, have a sibling living with chronic pain, and be able to speak and read English. Parent participants were required to have a child 8 to 18 years old living with chronic pain and be able to read and speak English. Research staff contacted eligible participants via e-mail to provide more information about the study, invite participation, and obtain informed consent. Participants who agreed to participate consented using an online consent form through Research Electronic Data Capture, a secure online data collection tool.^38^ The institutional research ethics board of the Hospital of Sick Children in Toronto approved the study (REB #1000070100) and participating sites in accordance with local requirements.

Interviews occurred within the second wave of the pandemic in Canada, between the months of September 2020 and March 2021. All interviews were conducted virtually by two female team members (T.K. and V.M.); the lead author (T.K.) is a research fellow with doctoral-level training in advanced qualitative methodology, and V.M. is a clinical research project coordinator who was trained in qualitative interviewing and supervised by the lead author. A semistructured interview guide was developed based on previous pandemic impact work^39^ and parallel qualitative work being completed with health care professionals who care for youth with chronic pain^40^ (see Supplementary Materials for the youth, sibling, and parent interview guides). The guides were based on key concepts from the family adjustment and adaptation response model^35^ (further described below) and focused on exploring youth and family member experiences throughout the COVID-19 pandemic and the impact on their pain, coping, and family life. The interview guides were pilot tested with four patient partners with lived experience and one parent partner with lived experience. Interviews were conducted using Zoom for Healthcare and were audio-recorded for verbatim transcription and analysis. Interviewers used prompts to encourage participants to expand on responses or provide additional detail (e.g., “Can you tell me more about that?” “What was that like for you?”); prompts were iteratively modified to incorporate new questions based on preliminary interview analysis. Interviews were conducted at one time point and ranged in length between 11 and 68 min (mean duration = 34 min). All participants received a CA$30 gift card honorarium for their participation.

### Theoretical Perspective

The design, data generation, and analytic process for this study were broadly guided by family stress and resilience theory, specifically, the family adjustment and adaptation response (FAAR) model as outlined by Patterson was used as a theoretical foundation.^35^ The FAAR model is a strengths-based approach, integrating family resilience and family stress theory with the overarching perspective that families are resilient in the face of various life challenges.^35^ This model is specifically focused on four central familial constructs: demands, capabilities, meanings, and adjustment or adaptation.^35,41^ Within this model, multiple levels of systems are considered, including individual family members, the family unit and subsystems, and the broader community and social environment. Importantly, this model distinguishes between *stressors*, which are crisis events that occur at a distinct time point (such as a natural disaster), and *family strains*, which exist over time and may be impossible to avoid or change (i.e., a chronic illness). The ability of a family to respond to a stressor is understood in the context of the multiple demands they are managing simultaneously; over time, families adjust or adapt by using capabilities from one level of the family system to meet demands at another.^35^ Two of the most prominent family resources are cohesion (e.g., bonds of unity running through family life) and adaptability (e.g., family’s capacity to meet obstacles and shift courses), which were used as key analytic concepts. Overall, this integrated model has been widely used as a theoretical foundation for exploring the relationships between families’ capacities, stressors, and demands and was especially relevant to guiding this exploration of how families living with chronic pain were able to face the challenges of the COVID-19 pandemic.^35,42^

### Data Analysis

A reflexive thematic analysis approach as outlined by Braun and Clarke^43,44^ was used to guide data analysis. Transcripts from the interviews were uploaded and analyzed using Dedoose software,^45^ a cross-platform cloud-based application for analyzing qualitative and mixed methods data.

Throughout data generation and early analysis, three team members (T.K., V.M., F.N.) read full transcripts to become familiar with the data and met weekly to reflexively conceptualize patterns and develop key concepts for the codebook. A combination of inductive and deductive approaches to code development was taken, where codes were generated inductively directly from the data using the language of participants, in combination with deductive use of key theoretical concepts from the FAAR model.

After the initial codebook was established, three team members (T.K., V.M., F.N.) coded two transcripts from each participant group (youth with pain, sibling, parent). Codes were compared and modified, and the codebook was restructured several times to account for new data as coding progressed. After coding 15 transcripts (5 from each group), the codebook was finalized and no new codes were being developed. Once all transcripts had been coded once, two team members (V.M., F.N.) reviewed all previously coded transcripts with the finalized codebook to ensure that all codes were captured. Following this, sets of excerpts or quotations for each code were reviewed and ranked according to how closely they reflected the “essence” of the code.^46^ Themes were then constructed by reviewing the exemplary excerpts and developing an account of the experience across all three perspectives (youth, sibling, parent). A team of coauthors (T.K., V.M., F.N., R.K., J.S.) reviewed the themes and contributed to the development of theme names and descriptions. Themes were finalized by writing up results and weaving participant data (quotations) with FAAR model theory into an analytic narrative that described the experience of living through the COVID-19 pandemic while managing chronic pain.^46^ Quotations from a range of participants were included to ensure that a variety of perspectives were represented across each theme.^46^

Data generation and analysis was conducted by researchers who hold a range of backgrounds (i.e., clinical, research, lived experience of chronic pain, etc.) and have interest in chronic illness management across the life span. Reflexivity was incorporated throughout the research process both individually and as a group; the team routinely discussed their relationship to and interest in the research topic and examined how their academic, professional, and personal positionality influenced the interpretation of data throughout the analytic process.

## Results

A total of 44 participants (14 parents, 19 youth with pain, and 11 siblings) from across Canada were purposively sampled to be interviewed from a group of *n* = 70 eligible. There were 42 transcripts in the final analysis because two audio recordings (1 parent, 1 youth) were poor quality and interviewer notes were used to supplement. Two triads, which included a parent, youth with pain, and their sibling from the same family, were interviewed. Demographic characteristics are reported by group in Tables 1 and 2; the largest group of participants was from Ontario (*n* = 22) and most participants identified as female (*n* = 34; see Tables 1 and 2).

**Table 1.** Characteristics of youth with chronic pain and their siblings.

Characteristics	Youth with chronic pain (*N* = 19)	Sibling^a^ (*N* = 11)
Age (mean, SD)^b^	16.5 (1.4)	16.4 (4.6)
Gender (*n*, %)		
Female	17 (89.5)	7 (70.0)
Male	2 (10.5)	3 (30.0)
Sex (*n*, %)		
Female	17 (89.5)	7 (70.0)
Male	2 (10.5)	3 (30.0)
Education (*n*, %)		
Elementary school (grades 4–8)	1 (5.3)	3 (30.0)
High school (grades 9–12)	13 (68.4)	4 (40.0)
Postsecondary (college/university)	4 (21.0)	1 (10.0)
Other	1 (5.3)	2 (20.0)
Ethnicity (*n*, %)^c^		
White/European	17 (85.0)	10 (77.0)
South Asian	1 (5.0)	2 (15.3)
Black	0	1 (7.7)
Other	2 (10.0)	0
Province of residence		
Alberta	6 (31.6)	1 (10.0)
British Columbia	1 (5.3)	0
New Brunswick	1 (5.3)	0
Nova Scotia	2 (10.2)	0
Ontario	9 (47.4)	9 (90.0)

^a^One sibling participant did not complete the demographic questionnaire, missing all demographic characteristics (*n* = 1).

^b^Missing for age (*n* = 2).

^c^Response option for ethnicity was “select all that apply.”

**Table 2.** Characteristics of parents.

Characteristics	Parents (*N* = 14)
Age (*n*, %)	
25–34	1 (7.1)
35–44	3 (21.4)
45–54	9 (64.3)
55+	1 (7.1)
Gender (*n*, %)	
Female	12 (85.7)
Male	1 (7.1)
Other (e.g., gender fluid, nonbinary)	1 (7.1)
Sex (*n*, %)	
Female	12 (85.7)
Male	2 (14.3)
Highest level of education (*n*, %)	
Secondary school/high school	2 (14.3)
College, technical school, or CEGEP	5 (35.7)
University	2 (14.3)
Postgraduate degree	4 (28.6)
None	1 (7.14)
Income (*n*, %)^a^	
Under $29,999	1 (7.60)
$30,000–$59,999	3 (23.1)
$60,000–$89,999	3 (23.1)
Over $90,000	3 (23.1)
Prefer not to answer	3 (23.1)
Province of residence (*n*,%)^b^	
Alberta	1 (7.6)
British Columbia	2 (15.4)
Nova Scotia	3 (23.1)
Ontario	5 (38.4)
Saskatchewan	2 (15.4)
Marital status (*n*, %)	
Single	1 (7.14)
Married or common law	10 (71.4)
Separated or divorced	3 (21.4)
Ethnicity (*n*, %)^c^	
South Asian	1 (7.7)
White/European	12 (92.3)
Living condition (*n*, %)	
Living with spouse	11 (78.6)
Living with child	11 (78.6)
Living with parent	1 (7.14)

^a^Missing for income (*n* = 1).

^b^Missing for province of residence (*n* = 1).

^c^Response option for ethnicity was “select all that apply.”

Qualitative results have been organized into three overarching themes that describe various aspects of the experience of families living with chronic pain throughout the pandemic. These three major themes are (1) absorbing and shifting: the toll of the pandemic on the family system; (2) social ambiguity and abandonment; and (3) building community resilience: familial adaptation to the pandemic. Each of these themes and subsequent subthemes are further described in depth below; please see Table 3 for additional quotations.

**Table 3.** Additional quotations.

Theme number	Theme/subtheme	Quotation
1.a.	Absorbing the loss of coping mechanisms	Usually with stress I have many, many ways that I know to deal with it, but it’s been quite limited, and I can’t seem to get away. And it’s made it a lot harder and it’s increased distress when you can’t get rid of it or calm it down—it builds up and it just impacts everything overall. With having nowhere to really go, it’s less motivation to go out and get that sunlight and get that like exercise and all that, and it’s just kind of all-totaling. (Youth 007)
1.b.	Shifting roles to respond to the pandemic	It’s more like how it’s [COVID] impacted my sister. She became really suicidal because of it, so that stressed me out even more. Which, well, when you’re stressed it ups your pain. So I’m definitely not sleeping because anytime my mom’s working night shifts, then I’m up until like 7:30 in the morning trying to make sure that she doesn’t do anything. I’m kind of glad that now it’s kind of like settled down a little bit. … So it’s maybe just affected my sister more? Which causes more stress on me. (Youth 020)
2.a.	Social sacrifice	I did have a couple friends stop talking to me period, that was just because I wasn’t hanging out with them because I wasn’t allowed to hang out with them because it was a thing. My parents didn’t want me seeing anybody. […] I have an older brother who lives in Saskatchewan and so we haven’t been able to see him in about a year because of the pandemic. So that’s more difficult on my family. (Youth 011)
2.a.	Social sacrifice	I know that a lot of people who are going out and getting the virus are people in my age demographic. Because for some reason a lot of these people, they seem to have the mentality it’s all okay, it won’t happen to them. And then they go around. And even if they themselves are asymptomatic, they’re still a carrier and they carry on to other people. And yes, it’s not good and it’s very irritating, especially ’cause I know people like that who are going out and socializing. And even if it’s not in huge groups, you’re still not supposed to do it. So it’s just irritating and in my head, even though I’m not personally doing it, I do in a way feel responsible. … (Sibling 008)
2.b.	Feeling abandoned by the health care system	They [health care team] thought I wasn’t as important as my case is, so it was a bit frustrating and … just made me feel not the best. They said that they were thinking I was completely fine, so it was hard, but I kept at them and making sure I got the appointments. (Youth 003)
2.b.	Feeling abandoned by the health care system	Because she’s had other symptoms develop, which probably now she has full-blown EDS, not just hypermobility spectrum disorder. That was part of the whole plan in the summertime was to reevaluate to see if there were criteria changed. So I don’t know what that’s gonna look like. It was initially just canceled outright and then finally got rescheduled. Like I said, it’s a Zoom call. Same thing with pain program—haven’t had any in-person appointments. Anything with the EDS clinic, ’cause she was going regularly, she would meet with the psychologist, she would meet with the physiotherapist and then the nurse practitioner as needed. That was canceled outright—she hasn’t been back since. (Parent 002)
2.b.ii.	Challenges with transition	The chronic pain clinic, really, they left it that he could contact them if he was having difficulty, but they, too, were trying to make that transition to adult care. So, no, the transition to adult care hasn’t happened smoothly in a sense that right now he’s in a strange gap. (Parent 014)
2.b.ii.	Challenges with transition	I’ve had a couple of ER [emergency room] visits which have actually been really hard because now that I turned 18 and with the pandemic, they don’t want to allow my mom to come in, which is understandable, but also because I just turned 18, like, there’s so much of my medical history that, like, I need my mom for. I’m not used to advocating completely for myself yet. […] So I do think that’s been a little tough because I have to go in—they don’t want my mom to come like even for regular appointments, so it’s been a little bit hard. (Youth 008)
3.a.	Family cohesion: Strengthening bonds in response to the pandemic	I got a lot closer to my family. We don’t tend to spend a whole lot of time with things like family time, ’cause we’re super busy. It has been nice to get a bit more of that. (Sibling 010)
3.b.	Confidence and self-management	Being able to be in a very comfortable environment where I can be where I want to be and then eat when I want to eat and sleep when I want to sleep and exercise when I want to exercise. All of that has also probably been beneficial for me. (Youth 016)

### Absorbing and Shifting: the Toll of the Pandemic on the Family System

The first major theme developed from this data related to the systemic toll of the pandemic on families. This all-encompassing impact changed many aspects of living with, and therefore coping with, chronic pain, and the implications of pandemic restrictions were demonstrated by the reports from youth that early in the pandemic their pain became worse:

The first initial shutdown when everyone was locked—not really locked but inside their homes and you only could like go out for groceries and stuff—that was, I’d say, the worst part of it. Because I was very sad, too, but nothing was really going on and I’ve seen the same people everyday and the pain just kept getting worse. (Youth 003)

This all-consuming effect of the pandemic extended beyond the youth living with pain and reverberated throughout the entire family system, as this sibling described:

I felt the stress and … I think his [sibling with pain] stress impacted my parents because they were concerned and then it kind of translated to stress for me. And I was just worried about him and the family. I just wanted to make sure that I was doing what I could to help even though I felt like there wasn’t much I could do. (Sibling 002)

This quotation demonstrates the way stressors, such as a global pandemic, are absorbed and distributed throughout the family unit and underscores how additional family strains (such as living with chronic pain) impact the collective navigation of the pandemic. One parent explained the way they experienced physical manifestations of pandemic-related anxiety and the resulting exacerbation of their mental health challenges:

My anxiety is through the roof because trying to juggle work and three medical kids plus my medical condition, because I do have an abnormal heart rhythm. […] My anxiety has been pretty high, and trying to keep that under control—even my depression has gone off a few times. And my PTSD [posttraumatic stress disorder] usually only affects me during the night for nightmares, but I have had two episodes where my PTSD actually affected me during the day, which has never affected me during the day until this pandemic. (Parent 007)

Challenges managing both pain and mood throughout the pandemic extended beyond the patients themselves and impacted families broadly as they moved through stages of the pandemic. Overall, this theme was characterized by participants absorbing the many changes to their daily lives, often related to the loss of coping mechanisms and strategies and shifting to adapt to the conditions of the pandemic.

#### Absorbing the Loss of Coping Mechanisms

Participants highlighted some of the key challenges of managing pain while living through the COVID-19 pandemic, specifically the interplay between pain and mood and the lack of distractions from pain during the height of pandemic restrictions:

It is different with the way I’m able to cope with it. It used to be that I would be able to locate the source of whatever was causing a really bad headache or really bad stomach pains and just kind of work from there. If it meant that I needed to change environment or have some time away from stress or different things. But now my only options are try to get to quiet, which is difficult when there’s, like, people and animals and stuff in the house and it’s the only place I can be. So it’s like my only options now are just to go to my bedroom and it’s very limited. It’s a lot more difficult to cope with it. (Youth 007)

This participant expressed the compounding effects of the pandemic on pain management, where not only were available coping mechanisms extremely limited but the ability to cope by “getting to quiet” within the home was also challenged by the presence of family members and pets. The way the pandemic limited strategies for pain management and caused a lack of distractions was a pervasive concern:

I think everything being online and not having a lot to do, especially no distraction, no nothing, that’s kind of made it worse. […] I just feel like you don’t have a lot to do or not enough distraction, especially over the summer where there’s no school. So you’re just kind of stuck inside doing nothing, which has a big impact. Which is surprising, ’cause the dream is to do absolutely nothing, but I think differently now. (Youth 009)

This youth described how the experience of the pandemic shifted their thinking, as they realized the true impact of doing “absolutely nothing” on their experience of pain. This was echoed by many patients who felt that limitations in their coping strategies impacted not only their pain but their overall motivation and routines (see Table 3).

Participants described a lack of opportunities for distraction (a previously established and reliable coping mechanism and source of stress relief) and the corresponding impact, as one parent explained:

It takes longer for him to get out of that funk when he gets the pain; he gets focused on the pain because there’s nothing to distract him really from it. (Parent 004)

Participants were cognizant of the interplay between pain and mood and described the cycle of the pandemic impacting mood and then a change in mood impacting pain. Beyond the youth themselves, this lack of distraction also impacted the way family members coped with managing a child with chronic pain, as one parent described the toll of witnessing her child’s constant suffering:

And there was no distraction for me and for him, right. So, whereas before I would go to work and at least have a break, I was watching him suffer all the time. And he was at home all the time, so it was almost more time to focus inward as opposed to being distracted by school or friends. (Parent 012)

This quotation demonstrates how the experience of pain challenged the family system as a constant strain on top of the stress of the pandemic, as families witnessed the suffering and absorbed the experiences of the person with pain that they may not have observed previously.

#### Shifting Roles to Respond to the Pandemic

Another key subtheme related to the way family roles shifted based on the demands placed upon the system; these demands were shaped by how family members who were struggling the most were doing. To balance the system, there was often a renegotiation of roles required or reprioritizing of one family member’s emotional or physical health over another’s:

I mean, my stress definitely has taken a toll ’cause it’s been every day—I feel like I’m on watch every day 24/7. […] There’s days where I have to put everything on hold that I’m doing and feeling and just say, “We need to just deal with the day and the hour here. …” I mean as a parent, I can’t just drop her off at somebody’s house so I can see if I need to go to the doctor myself. I know I put off my own mental health. … I know I have to put stuff off because there’s days where I can’t bring her somewhere ’cause she’s beyond herself, actually in pain. (Parent 006)

This parent’s experience illustrates how the family’s attention had to be focused on the child with pain, at the expense of looking after their own health concerns. This sentiment was also echoed within a sibling relationship (see Table 3).

These quotations demonstrate how families function as multisystem units, constantly shifting to ensure the members who require support are taken care of. In this example, the participant took on a caretaking role, supervising her sibling while their mother was working overnight. This type of caregiving was often exacerbated during the pandemic, when access to health care providers, community services, and friendships was limited, requiring family members to increase their caregiving work:

But other days, she’s just in so much pain she can’t even hold her head up like, she just—she’s just laying down on her heating pad ’cause that mildly helps and she just hurts and I feel so awful. And, you know, I’m like, “Hey do you want tea?” “Hey, do you need some pain meds?” I feel really awful and I know it sucks to hurt, like, I can only imagine. (Sibling 008)

Siblings emerged as a key source of support through caregiving, providing “distraction, comfort and companionship” (Parent 003). Siblings often provided tangible support with activities of daily living (e.g., hair washing, laundry, groceries: Sibling 008), pain management support (e.g., pain medication: Sibling 008; massage, deep breathing: Parent 003), and emotional support and company (visiting in hospital: Sibling 013; watching TV together: Sibling 008). A quotation from one parent highlighted this important caregiving connection between her children:

She’ll [sibling] do deep breathing and then he’ll [child with pain] do deep breathing back. So if you can imagine every sort of physio or mental health session or OT or anything, she’s been at all of those appointments alright—she’s like a mini practitioner of everything! Yeah, she is not licensed, but she has her own technique. … She has “paws” and she has “chopping sushi”—they’ve named them. Yeah, [sibling] can, like, see one crease on his face or the position of his eyebrow and she knows exactly what’s going on and what he likes—it’s unbelievable. (Parent 003)

This parent described the physical acts of caretaking provided by her child to help her sibling manage their pain. Overall, this theme was characterized by the ways that families shifted and adjusted during the pandemic, often absorbing additional or new caretaking responsibilities to respond to the needs of family members who required the most support, at a time when many additional health services and coping mechanisms were limited.

### Social Ambiguity and Abandonment

The second major theme developed from interview analysis explored participants feeling disregarded by society and by the health care system that cared for them; specifically, by witnessing their peers and social circles ignore COVID-19 precautions while they were making major social sacrifices:

We all kind of agreed that this was the only close circle—we weren’t going to parties, we weren’t hanging out with other people, like, we’re just trying to be as careful as we could for teenagers. There was a kid last weekend who had a party and then their sister had COVID and then now, like, all that group is isolated. I just shake my head at some people—I don’t understand why they’re having so many people come over, and I don’t get that. Because they’re complaining about COVID, yet they’re doing things like that, which is not helping whatsoever, and it’s just like—it just kind of grosses me out because everyone would love for this COVID to disappear and go move on, but it’s not going to happen when people don’t understand the severity of it. (Sibling 002)

Participants discussed how they negotiated risk and made decisions within their family systems to agree upon collective action to limit exposure to COVID-19. This social ambiguity was generally experienced as a feeling of unease as participants witnessed peers who lacked an understanding of their family as vulnerable due to their health care conditions or refused to take COVID-19 precautions, as well as feeling abandoned by the health care system and their providers who normally support them during challenging times such as health crises and transitional periods.

#### Social Sacrifice

This subtheme of social sacrifice was characterized by a sense of grief and loss, as participants limited their social activity and, as a result, often missed out on major milestones marking important transitional periods (such as school graduations, starting postsecondary school, or moving out of their family home). Participants felt they were making significant sacrifices such as staying at home, wearing masks, and limiting visits with friends and loved ones while they witnessed others who continued to act like “nothing was going on” (Parent 013) and not “follow the rules” (Parent 002):

If the restrictions get a little bit harder, then we’re willing to sacrifice some of our mobility, if we can make sure that the numbers of people getting the virus goes down. We’re all pretty self-contained, it’s not a big issue. Just, some of our concerns—we have friends that still go out like nothing’s going on. So what we’ve done is we’ve kind of distanced ourselves from them. (Parent 013)

Participants expressed the mental energy required to constantly witness people rejecting public health measures, which seemed to put them at risk. As the pandemic progressed and restrictions lessened, some participants described how they did not feel safe in various environments as part of a family living with someone vulnerable:

I think the problem for me and my family is that the regulations aren’t strict enough—we don’t really feel safe going out anymore. (Sibling 010)

Beyond this, participants described the major losses and grief that accompanied living through the pandemic as they missed opportunities to see family, celebrate milestones together, and mark life transitions.

Many participants described the way their social circles changed because of the inability to socialize or having to cut off relationships with people who were ignoring COVID-19 precautions. This ability to ignore the rules without consequence was especially challenging for young participants and highlighted the social ambiguity of the pandemic situation:

Personally, I stopped hanging out with some of my friends or people I thought were friends because they’re the ones going out and partying. You see the true colors of who people are, and I’m not okay with that kind of—to be honest—selfishness. So I would rather not deal with them. […] You see the people who are going in partying, and they’re posting on social media, completely blatant, and they’re not getting consequences, right? And then everything is fine. […] It makes you feel affected because there are no consequences. (Sibling 011)

Because of the age group of the youth in this study, many participants were in high school or were young adults and witnessed their peers ignore public health restrictions to socialize. This left participants in a situation where they missed out on important moments of socializing or feeling responsible for the fact that it was their age group that was spreading the virus (Sibling 008).

Overall, this theme was characterized by participants feeling they had entered a social contract that was not being upheld consistently by their peers and communities, and they felt misunderstood or left to fend for themselves during a period of extreme stress and isolation.

#### Feeling Abandoned by the Health Care System

Participants also expressed feeling abandoned by the health care system or their health care teams during a time when they required more intensive care or support due to the challenges of the pandemic. This subtheme was distinguished by feeling that their health care providers or teams were unreachable or unresponsive and not always available to support patients with their pain management:

The biggest concern is just being able to actually access help when we need it. Because instead of being able to call the doctor and actually talk right away to secretary or stuff, there’s been times where I don’t get an answer for two weeks. And it’s just really—they’re answers that we need, and how do you tell your kid, “I’m trying?” But it’s like … “I have no answers for you.” (Parent 006)

Beyond challenges contacting providers and making appointments, participants’ access to community-based health care and services that they used to manage pain prior to the pandemic was very limited:

The massage therapist and my chiropractor shut down, so I couldn’t go see them, and then the only one that I really talked to throughout the pandemic was my psychologist. So she was very helpful, but, like, the physio part wasn’t there and the rest of the stuff, so it was—there’s a lot more pain going on than usual. (Youth 003)

Participants described the impact of this lack of access to care, specifically how an inability to see providers such as physiotherapists, massage therapists, chiropractors, etc., contributed to worsening pain over the course of the early pandemic. There was also a sense that the health care system, or some providers, did not recognize the severity of some cases, which contributed to a sense of abandonment. The switch to using primarily virtual care also emerged as a challenge for some participants. Whether they were working with providers they knew or meeting a new team, they reported having challenges forming connections (Youth 008; Parent 012) or trying to get care for their children who did not “like video conferencing” (Parent 004):

In terms of the chronic pain, it was actually a horrible experience. He got notice that he would need an MRI and a scope right around March 13th, when everything shut down. And so everything was kind of on hold. A lot of the doctors at the pain clinic we only saw online, which was hard to connect at times and hard to get to know the doctors and have the doctors get to know us. (Parent 012)

This systemwide shut down of in-person health care limited access across a variety of settings, resulting in participants experiencing delays in care and challenges transitioning out of the pediatric health care system.

#### Delays in Care

Families reported experiencing significant delays in care. Whether receiving diagnostic tests (Youth 008 and Youth 003) and referrals (Parent 004), facing medication shortages (Parent 009), or waiting for other support for pain management, participants described how their usual access to health care providers became limited or completely inaccessible:

See that’s where I think there might be a delay. Because he was referred to an adult headache neurologist—that was well over a year ago now, maybe even a year and a half ago and we still haven’t heard. So that might be slower because of COVID. (Parent 004)

I was supposed to have an MRI and then it got pushed like very far. […] Then they said that they never received a requisition for it, so then they had to send a new one, so it took a lot longer than it would have to get in to see them. But other than that it was mainly just the appointments that would get canceled and not always scheduled the same. (Youth 003)

Participants expressed the challenges they faced with appointments and tests being extremely delayed or visits being canceled outright; in the case of one youth participant, their condition seemed to progress and worsen, which they felt was connected to waiting so long for the results:

I have a lot of new issues and I’ve had two knee surgeries and I need another surgery, but it was hard because they couldn’t assess my knees to actually see what was happening. My MRI was postponed for, like, months and months because obviously they weren’t number one priority and they were limiting who would like us to come into the hospital. So things were kind of postponed, and then once I finally got the images they were like, “Oh, this issue is worse than we thought,” because I ended up waiting so long to get to that. (Youth 008)

Overall, participants experienced a variety of consequences related to changes and limitations to health care access, and delayed services contributed to challenges with pain management and beyond.

#### Challenges with Transition

Many participants in this study were in the process of transitioning from pediatric to adult care settings throughout various points of the pandemic. This transition period is already known to be challenging for patients with chronic illnesses,^47^ but the pandemic seemed to exacerbate preexisting issues related to transfer of care:

It was quite difficult because I turned 18 right in the midst of the pandemic, so it was really hard because there were a lot of gaps in my health care. Things weren’t transitioned over properly, because we weren’t going in person. So I turned 18 in June, then I didn’t have some of my appointments until September. So that gap was like, “Oh, what am I supposed to do?” We needed other doctors to refill medications and I wasn’t getting the same treatment, so it was hard. (Youth 008)

Both Youth 008 and Parent 014 highlighted the “gap” in care they experienced during the transitional period and how the transition did not “happen smoothly” because of the pandemic and lack of in-person care. This concern regarding managing transitions through virtual care was also highlighted by this parent:

With the adult EDS [Ehlers Danlos Syndrome] clinic, they were supposed to do it in the summertime—there was supposed to be a reevaluation at that time and have the person from the adult clinic present over at [Children’s Hospital] to help with that transition process. That was completely canceled, couldn’t even get answers from people as to: “Are we going to re-book this in the fall?” I finally did get a response back that they’re doing it as a Zoom call. We have that for next week. But the whole physical part is missing. How are they going to reevaluate her? (Parent 002)

Participants facing transitions reported that either their transitional care moved to a virtual setting, which may have been insufficient for the necessary assessments, or the care participants were supposed to receive “completely stopped” (Sibling 010) and they felt as though no one was responsible for looking after them, as this youth explained:

Participant: At the beginning of the pandemic, I was doing really, really well, ’cause I just got out the hospital and, like, everything was really well managed—at the beginning I was the best that I’ve been in years.

Researcher: And did that kind of stay consistent throughout from now to March? Has that been consistent?

Participant: No, because things just have not been taken care of; for example, my feeding tube. … I also just switched from pediatrics to the adult world, too, so that was another big issue that nobody was taking care of things and taking responsibility for things. So things just really went downhill, and nobody really knew what to do. (Youth 004)

Beyond coordinating the transition, participants also discussed how the COVID-19 pandemic impacted their experience of care in the new setting; in this case, one participant described how they had to attend appointments alone because of hospital policy in the adult care setting.

Overall, this theme described a sense of interruption in the transition period, leaving participants feeling unsure about where they fit within the system and who to contact to manage their care.

### Building Community Resilience: Familial Adaptation to the Pandemic

This final major theme illustrates how families adapted and adjusted to the pandemic using their existing strengths or resources and often developed new capabilities and ways of understanding or responding to the stress of the pandemic:

I think it’s allowed us to come back together in a way—we are literally together all the time. And to build community resilience. So there are points where I’ve been really resilient and [child] hasn’t been or [child’s sister], but now we’re sort of building a herd immunity of resilience. (Parent 003)

This parent focused on how their family built resiliency through the pandemic, explaining how their resiliency shifted fluidly but also likened it to “herd immunity,” where all members involved rely on each other to bolster their own resilience. Another participant echoed this sentiment:

I definitely feel closer with my parents. […] You realize how our family can really come together and support each other, and I really appreciated that; it was really nice to see. I can only imagine the stress that my parents were going through with one child with pain and another with mental pain and just trying to navigate it for themselves as well. I really appreciate it, how well they were able to handle it and how accommodating they were with the situation. But, yeah, I definitely learned a lot of adaptiveness with myself. (Sibling 002)

This quotation highlights the combination of gratitude for family cohesion and a sense of adaptiveness and building confidence, two key components of this theme that will be explored in further depth.

#### Family Cohesion: Strengthening Bonds in Response to the Pandemic

In this theme, the pandemic emerged as a relationship strengthener. Although some families reported increases in conflict or irritation due to close quarters, many highlighted how the pandemic created an environment that enhanced or strengthened their relationships within their family unit:

I’ve definitely, definitely gotten closer to my family. I mean, sometimes, it’s kind of a lot, being home with them, especially when my brothers were home all day and I was home all day, it was a lot then, but it definitely has been nice in some ways. (Sibling 009)

This sense of family cohesion seemed to develop or strengthen in direct response to the stressor of the pandemic and associated restrictions that were placed on families. Family cohesion, defined by Patterson as “the bonds of unity running through family life” is considered one of the most prominent family resources.^35^^(p216)^ In the case of the pandemic, this unity was related to families’ ability to collectively cope with the challenges they faced:

I guess for some people it’s almost like a bonding experience. ’Cause it kind of tests relationships, both romantic and familiar relationships, ’cause I’m stuck with these people or this person for months on end. So that kind of tells you, like, what your breaking point is. There are moments where people obviously get a little snippy and they got cabin fever and they’re irritable, and they can’t go outside and do fun things so they’re irritated about that and everybody gets on each other’s nerves every once in a while. So I feel like this can, in a way, strengthen relationships, so that’s cool. It’s like we survived the pandemic together. (Sibling 008)

This participant described how the pandemic acted as a “bonding experience” for her family and they came out triumphant having survived together. This type of family cohesion was also demonstrated through how decisions were made together, based on what was best for the family as a complex system:

Considering how close the grocery store is to our house, and I have the strongest immune system in my family, and I’m the most capable physically in my family. So I would just go down the block to the grocery store and I did all the grocery pickups. So anytime we need groceries, I’m the one who goes and runs out and gets it. (Sibling 008)

This strong sense of family cohesion and unity was one of the ways in which families were able to enact their adjustment to pandemic life and move through the variety of challenges they experienced during this time.

#### Confidence and self-management

Another key resource or capability that was built in response to the pandemic was increased self-confidence. This increase in confidence was mainly reported by the youth who were living with pain and was a prevalent theme throughout many interviews when exploring pain management during the pandemic:

I feel like it’s probably increased my confidence because I’ve had so much time to try and figure it out and I’ve been able to find coping mechanisms that work. So an attitude toward it is definitely changed. (Youth 015)

Youth described how the pandemic provided space and “time to try and figure it out” (Youth 015) and that because of this, they developed increased confidence (Youth 015, Youth 018, Youth 003). In addition, the limited access to health care providers and changes in routine provided them an opportunity to engage in more pain self-management behaviors than they may have explored otherwise:

Well, before the pandemic I was not … well, I was confident but not as much as I am now. It’s definitely brought me to be more confident with managing my pain. And I’ve learned other ways to help with that, too. I really self-manage a lot more than I did before. I usually would talk to the doctors a lot more and ask them what I should do to help it or what they think is some good, helpful things to do. … It was harder to reach them [the health care team during the pandemic] and so I just kind of took it in my own hands and tried some different things and found what worked best. (Youth 003)

This youth described how they were able to take pain management into their “own hands” and try out various strategies; other participants discussed how they were able to incorporate techniques such as meditating (Youth 010) and exercise (Youth 018) more easily into their routines at home:

Well, one positive thing I guess would be I started meditating more ’cause I had more time ’cause I was at home, and at school I wouldn’t really say, “Hold up a minute, let me meditate.” (Youth 010)

Participants overall witnessed some positive aspects of pandemic restrictions, primarily related to the flexibility of schedule that allowed them more control over their routines, which often led to improvements in pain.

Overall, youth living with pain reported that they experienced noticeable increases in their self-confidence and their ability to self-manage their own pain during the pandemic, which were related both to having more separation or independence from their care teams and to having more flexibility to implement self-management strategies into their lives.

## Discussion

This qualitative study examined the experience of the COVID-19 pandemic for families living with chronic pain and analyzed perspectives of patients, siblings, and parents together. Our results highlighted three main themes: (1) absorbing and shifting: the all-consuming toll of the pandemic on the family system; (2) social ambiguity and abandonment; and (3) building community resilience: familial adaptation to the pandemic. Our findings highlight the unique experiences of families living in Canada navigating the challenges of the pandemic while also coping with chronic pain and the additive impact of various public health measures (i.e., social distancing, limiting in-person health care, school closures) on this population.

The pandemic had a direct impact on youth with chronic pain and their families. Youth expressed changes in pain symptoms and mental health challenges, which subsequently impacted their caregivers and siblings, resulting in changes to family dynamics. Some quantitative studies among youth with chronic pain reported no change^29^ or reductions in pain symptoms during the pandemic^21^; however, many studies found increases in mental health challenges.^21,29,48^ Our research team completed a large survey-based study with over 600 youth, siblings, and parents and found significant increases in mental health symptoms such as anxiety, depression, and PTSD; however, significant changes in pain intensity were not found.^37^ In contrast, most qualitative studies among youth and adults with chronic pain recounted a deterioration of their pain condition during the pandemic.^22,49^ This is likely due to the interplay of pain and mood (also described in our analysis) and is supported by literature that suggests a bidirectional relationship between pain and mental health due in part to their shared neural mechanisms.^50^

In the context of the pandemic, it has been hypothesized that uncertainty and lack of control can increase anxiety, distress, and depression, which can make coping with pain more difficult.^51^ Such changes in pain experience may hinge on an individual’s personal experience with the pandemic and their ability to adapt to it, resulting in some positive and some negative experiences. For example, increases in accessibility and remote school options may allow youth to experience a more flexible environment; our results demonstrate how this flexibility allowed youth to build more self-management practices into their routines. In contrast, the sudden and large-scale shutdown of in-person clinical and community services resulted in difficulties accessing care, and many of these establishments were primary tools of pain management for these youth. Moreover, for youth with chronic pain, whose peer support systems are smaller compared to youth without pain,^52^ the pandemic may have isolated and reduced their peer support systems further, increasing mental health challenges. This required parents and siblings to take a more active role as social support for youth with chronic pain. Prior to the pandemic, parents and siblings were already taking on additional caregiving roles, typically at the expense of their own physical and mental health.^53–57^ The pandemic exacerbated these experiences by adding additional demand that further strained the family system.

Participants, including youth with chronic pain and their family members, felt disregarded by society, including a sense of social ambiguity and abandonment by the health care system. This theme is consistent with previous literature surrounding the idea that real or perceived risk of infection can influence adherence to preventative measures.^58^ Adolescents’ and young adults’ risk perception and adherence to preventive measures are shown to be motivated by social responsibility and the desire to protect family and other vulnerable individuals.^58,59^ This concern for family safety could then potentially lead to frustration, anger, and resentment toward those ignoring COVID-19 precautions. Several studies have also identified disruption to the health care system as a direct result of the COVID-19 pandemic, specifically focusing on the impacts of triaging or prioritizing acute physical needs,^24^ leading to individuals with chronic pain experiencing delayed access^60–63^ and increased anxiety, helplessness, and pain.^24^ These findings highlight the importance of ensuring that individuals with chronic conditions continue to have stable access to various therapies even amidst public health crises.^64^ Utilization of virtual telemedicine can help to improve access for some individuals,^65,66^ but such online interventions must include evidence-based, multidisciplinary pain management supports^64^ and, as highlighted in our analysis, virtual care may not always be a suitable alternative.

Although the COVID-19 pandemic created many barriers for youth living with pain and their families navigating the health care system, families found ways to adapt to their new reality and emerged with a sense of resilience and cohesion. This is consistent with current literature that focuses on posttraumatic growth after experiencing adversity.^67^ Posttraumatic growth involves building resilience and thriving when faced with adverse experiences and is supported by ordinary adaptive systems such as relationships with family and peers, community support, and opportunities to succeed and build self-efficacy.^68^ In the present study, youth with pain and their families reported increased self-efficacy and confidence in self-management skills as the pandemic progressed, which aligns with current literature in other populations with chronic disease.^69–72^ Chronic pain self-management techniques include learning skills to self-regulate, adapting to changing levels of ability, and continuing to persist in the face of pain, and individuals who had self-management skills coped better with their condition during the pandemic.^70,71,73^

Finally, some studies have demonstrated that adolescents and youth experienced positive coping and higher subjective well-being during the pandemic, which was related to increases in emotional self-efficacy and self-regulation skills.^74^ Increased self-efficacy in pain self-management is related to improvements in pain interference, depressive symptoms, and health-related quality of life.^69,75^ Previous studies have also demonstrated that youth living with pain relied heavily on their family support system throughout the pandemic, and parents and siblings played a key role in building and enhancing the resilience of youth living with pain.^76,77^ Current research on COVID-19 and family cohesion is consistent with the sentiment expressed by families in this study. Family cohesion was also related to daily practices of gratitude, healthy communication, and bonding through positive activities that built a sense of togetherness and trust.^78^ Overall, family well-being and resilience were facilitated by adaptive coping and responding to adversity together, which aligns with the experiences of the families in this study.

## Study Limitations and Strengths

There are several limitations to consider while interpreting the findings from this study. First, this study used a maximum variation sampling strategy; however, participants were selected from those who had opted in from a larger online survey that was made up of predominantly White, educated, high-socioeconomic-status Canadians. This sample was lacking representation from racialized and structurally marginalized groups such as Black and Indigenous youth and families, which limits the transferability of results. Knowing that these groups may have been more severely affected by the pandemic,^79,80^ future research should focus on exploring their experiences. Second, interviews were conducted at one time point and therefore the findings reflect a certain phase of the pandemic, specifically prior to widespread vaccination in Canada. Also, although public health restrictions varied regionally, our data were not analyzed by region or province and there may have been differences in how participants experienced the pandemic in different locations in the country. Thirdly, although there were two triads of participants from the same family in this study, this was not an inclusion requirement. Going forward, it may be beneficial to focus on recruiting at the family level or conducting a case study of a smaller number of families (especially those uniquely or severely affected by the pandemic) to explore the impact of the pandemic on the family unit as a unique system.

One of the strengths of this study lies in the multiple perspectives that were analyzed collectively. This is the first study, to our knowledge, that includes the perspectives of youth, siblings, and parents, all analyzed together, to present a cohesive overview of the pandemic experience. Another strength of this work is the range of the participant sample, which included families from across Canada and an array of youth who were at various life stages during the pandemic. Furthermore, the study captured in real time the experiences of youth living with chronic pain and their families during a major public health crisis, which will be helpful to inform the response of health care systems to better support these families in the future, during this pandemic and beyond. Finally, our rigorous coding and analysis process was grounded in a solid theoretical foundation, which strengthens the quality of the results and enhances our ability to move beyond a descriptive account and provide a substantive report of the importance of these findings.

## Conclusion and Future Directions

Youth, parents, and siblings all reported that the COVID-19 pandemic affected their families in a variety of ways, and pandemic-related restrictions impacted coping strategies across the family system. These results outline the challenges that youth with chronic pain and families experienced with their overall health conditions throughout the pandemic and highlights the resilience that was built within families during this time. Our results demonstrate the importance of family relationships in absorbing the hardships of the pandemic and the cohesion that was developed because of increased reliance on family members. The study results also highlight the social consequences of living through a pandemic with a chronic health condition, yet also shed light on the positive aspects of this experience, such as the opportunity to develop and expand confidence in pain self-management and coping skills.

Future work should employ an intersectional lens to examine how racialized or structurally marginalized youth with chronic pain and their families experienced the pandemic, because they may have been disproportionately affected by various public health restrictions and by COVID-19 illness itself. In addition, it would be beneficial to explore the dynamics of the family unit in further depth, through in-depth family-based case studies, to identify specific targets for future intervention. Finally, future research should also focus on examining how benefits of the pandemic (e.g., increased confidence, increased self-management) may be sustained for youth with chronic pain and their families to best support these families into the future.

## Data Availability

The data that support the findings of this study are available from the corresponding author upon reasonable request.

## Appendix

Appendix

## References

[1] Campbell F, Hudspith M, Choinière M, El-Gabalawy H, Laliberté J, Sangster M, Swidrovich J, Wilhem L. An action plan for pain in Canada. Ottawa; 2021. doi:10.1787/9789264268661-8-en. PMC11290766 39086837

[2] Hogan ME, Taddio A, Katz J, Shah V, Krahn M. Incremental health care costs for chronic pain in Ontario, *Canada*: a population-based matched cohort study of adolescents and adults using administrative data. Pain. 2016;157(8):1626–18. doi:10.1097/J.PAIN.0000000000000561. 26989805

[3] King S, Chambers CT, Huguet A, MacNevin RC, McGrath PJ, Parker L, MacDonald AJ. The epidemiology of chronic pain in children and adolescents revisited: a systematic review. Pain. 2011;152(12):2729–38. doi:10.1016/j.pain.2011.07.016. 22078064

[4] Liao ZW, Le C, Kynes JM, Niconchuk JA, Pinto E, Laferriere HE, Walters CB. Paediatric chronic pain prevalence in low- and middle-income countries: a systematic review and meta-analysis. EClinicalMedicine. 2022;45. doi:10.1016/J.ECLINM.2022.101296/ATTACHMENT/9CCFBE46-9064-4008-B0AD-FB5B12DE3647/MMC4.DOCX. PMC8850335 35198925

[5] Canadian Pain Task Force. An action plan for pain in Canada. Ottawa (ON); 2021. [accessed 2022 Oct 2]. https://www.canada.ca/content/dam/hc-sc/documents/corporate/about-health-canada/public-engagement/external-advisory-bodies/canadian-pain-task-force/report-2021-rapport/report-rapport-2021-eng.pdf

[6] Campo JV, Bridge J, Lucas A, Savorelli S, Walker L, Di Lorenzo C, Iyengar S, Brent DA. Physical and emotional health of mothers of youth with functional abdominal pain. Arch Pediatr Adolesc Med. 2007;161(2):131–37. doi:10.1001/ARCHPEDI.161.2.131. 17283297

[7] Guite JW, Lobato DJ, Shalon L, Plante W, Kao BT. Pain, disability, and symptoms among siblings of children with functional abdominal pain. J Dev Behav Pediatr. 2007;28(1):2–8. doi:10.1097/DBP.0B013E3180307C26. 17353724

[8] Coffelt TA, Bauer BD, Carroll AE. Inpatient characteristics of the child admitted with chronic pain. Pediatrics. 2013;132(2). doi:10.1542/PEDS.2012-1739. 23821701

[9] Noel M, Groenewald CB, Beals-Erickson SE, Gebert JT, Palermo TM. Chronic pain in adolescence and internalizing mental health disorders: a nationally representative study. Pain. 2016;157(6):1333. doi:10.1097/J.PAIN.0000000000000522. 26901806 PMC4939835

[10] Tegethoff M, Belardi A, Stalujanis E, Meinlschmidt G. Comorbidity of mental disorders and chronic pain: chronology of onset in adolescents of a national representative cohort. J Pain. 2015;16(10):1054–64. doi:10.1016/J.JPAIN.2015.06.009. 26168877

[11] Valrie CR, Bromberg MH, Palermo T, Schanberg LE. A systematic review of sleep in pediatric pain populations. J Dev Behav Pediatr. 2013;34(2):120–28. doi:10.1097/DBP.0B013E31827D5848. 23369958 PMC3562475

[12] Battaglia M, Quinn PD, Groenewald CB. Consideration of adolescent pain in responses to the opioid crisis. JAMA Psychiatry. 2021;78(1):5–6. doi:10.1001/JAMAPSYCHIATRY.2020.1694. PMC7891903 32936227

[13] Groenewald CB, Law EF, Fisher E, Beals-Erickson SE, Palermo TM. Associations between adolescent chronic pain and prescription opioid misuse in adulthood. J Pain. 2019;20(1):28–37. doi:10.1016/J.JPAIN.2018.07.007. 30098405 PMC6309740

[14] Huguet A, Miró J. The severity of chronic pediatric pain: an epidemiological study. J Pain. 2008;9(3):226–36. doi:10.1016/J.JPAIN.2007.10.015. 18088558

[15] McMahon M, Nadigel J, Thompson E, Glazier RH. Informing Canada’s health system response to COVID-19: priorities for health services and policy research. Healthc Policy. 2020;16(1):112. doi:10.12927/HCPOL.2020.26249. 32813643 PMC7435075

[16] Mukhida K, Stewart J, Mehrpooya R, Fraser J. Virtual care for patients with chronic pain and addictions during the COVID-19 pandemic. Can J Pain. 2020;4(1):179. doi:10.1080/24740527.2020.1785856. 33987497 PMC7951171

[17] Poletti M, Raballo A. Coronavirus disease 2019 and effects of school closure for children and their families. JAMA Pediatr. 2021;175(2):209–10. doi:10.1001/JAMAPEDIATRICS.2020.3586. 33226404

[18] Stewart SL, Vasudeva AS, van Dyke JN, Poss JW. Following the epidemic waves: child and youth mental health assessments in Ontario through multiple pandemic waves. Front Psychiatry. 2021;12:1987. doi:10.3389/FPSYT.2021.730915/BIBTEX. PMC8635704 34867522

[19] Eccleston C, Blyth FM, Dear BF, Fisher EA, Keefe FJ, Lynch ME, Palermo TM, Reid MC, de C. Williams AC. Managing patients with chronic pain during the COVID-19 outbreak: considerations for the rapid introduction of remotely supported (eHealth) pain management services. Pain. 2020;161(5):889–93. doi:10.1097/J.PAIN.0000000000001885. 32251203 PMC7172975

[20] Nieto R, Pardo R, Sora B, Feliu-Soler A, Luciano JV. Impact of COVID-19 lockdown measures on Spanish people with chronic pain: an online study survey. J Clin Med. 2020;9(11):3558. doi:10.3390/JCM9113558. 33167322 PMC7694344

[21] Birnie KA, Kopala-Sibley DC, Pavlova M, Nania CG, Bernier E, Stinson JN, Noel M. The impact of the COVID-19 pandemic on youth with chronic pain and their parents: a longitudinal examination of who are most at risk. Children. 2022;9(5):745. doi:10.3390/CHILDREN9050745. 35626922 PMC9139609

[22] Dassieu L, Pagé MG, Lacasse A, Laflamme M, Perron V, Janelle-Montcalm A, Hudspith M, Moor G, Sutton K, Thompson JM, et al. Chronic pain experience and health inequities during the COVID-19 pandemic in Canada: qualitative findings from the chronic pain & COVID-19 pan-Canadian study. Int J Equity Health. 2021;20(1):1–13. doi:10.1186/S12939-021-01496-1/FIGURES/1. 34162393 PMC8220113

[23] Kaczynski KJ, Chang CYH, Chimoff J, Koike C, Berde CB, Logan DE, Nelson S, Kossowsky J. Initial adjustment to the COVID-19 pandemic and the associated shutdown in children and adolescents with chronic pain and their families. Front Pain Res. 2021:69. doi:10.3389/FPAIN.2021.713430. PMC8915775 35295442

[24] Villegas-Echeverri JD, Carrillo JF. Navigating the COVID-19 waters with chronic pelvic pain. Int J Gynaecol Obstet. 2020;151(2):172–74. doi:10.1002/IJGO.13359. 32936448 PMC9087540

[25] Snapiri O, Rosenberg Danziger C, Krause I, Kravarusic D, Yulevich A, Balla U, Bilavsky E. Delayed diagnosis of paediatric appendicitis during the COVID-19 pandemic. Acta Paediatr. 2020;109(8):1672–76. doi:10.1111/APA.15376. 32460364 PMC7283758

[26] Poppert Cordts KM, Stone AL, Beveridge JK, Wilson AC, Noel M. The (Parental) whole is greater than the sum of its parts: a multifactorial model of parent factors in pediatric chronic pain. J Pain. 2019;20(7):786–95. doi:10.1016/J.JPAIN.2019.01.004. 30658175 PMC6626577

[27] Weissman MM, Warner V, Wickramaratne P, Moreau D, Olfson M. Offspring of depressed parents. 10 years later. Arch Gen Psychiatry. 1997;54(10):932–40. doi:10.1001/ARCHPSYC.1997.01830220054009. 9337774

[28] Lowe SR, Godoy L, Rhodes JE, Carter AS. Predicting mothers’ reports of children’s mental health three years after hurricane katrin. J Appl Dev Psychol. 2013;34(1):17. doi:10.1016/J.APPDEV.2012.09.002. 23471125 PMC3587107

[29] Law EF, Zhou C, Seung F, Perry F, Palermo TM. Longitudinal study of early adaptation to the coronavirus disease pandemic among youth with chronic pain and their parents: effects of direct exposures and economic stress. Pain. 2021;162(7):2132–44. doi:10.1097/J.PAIN.0000000000002290. 34050112 PMC8205975

[30] Singletary B, Schmeer KK, Purtell KM, Sayers RC, Justice LM, Lin TJ, Jiang H. Understanding family life during the COVID-19 shutdown. Fam Relat. 2022;71(2):475–93. doi:10.1111/FARE.12655. 35600938 PMC9111772

[31] Martinez B, Pechlivanoglou P, Meng D, Traubici B, Mahood Q, Korczak D, Colasanto M, Mahant S, Orkin J, Cohen E. 53 health outcomes of siblings of children with chronic health conditions: a systematic review and meta-analysis. Paediatr Child Health. 2021;26(Supplement_1):e38–e39. doi:10.1093/PCH/PXAB061.042. 35810772

[32] Schinkel MG, Chambers CT, Hayden JA, Jordan A, Dol J, Higgins KS. A scoping review on the study of siblings in pediatric pain. Can J Pain. 2017;1(1):199–215. doi:10.1080/24740527.2017.1399053. 35005355 PMC8730589

[33] Nguyen L, Davis H, Bellefeuille S, Havens J, Jack SM, Di Rezze B, Ketelaar M, Gorter JW. Canadian resources for siblings of youth with chronic health conditions to inform and support with healthcare management: a qualitative document analysis. Front Rehabil Sci. 2021;52. doi:10.3389/FRESC.2021.724589. PMC9397918 36188805

[34] Gorodzinsky AY, Davies WH, Tran ST, Medrano GR, Bernacki JM, Burks LM, Anderson Khan K, Hainsworth KR, Weisman SJ. Adolescents’ perceptions of family dynamics when a sibling has chronic pain. 2013;42(4):333–52. doi:10.1080/027396152013842460.

[35] Patterson JM. Families experiencing stress. I. The family adjustment and adaptation response model. II. Applying the FAAR model to health-related issues for intervention and reasearch. Fam Syst Med. 1988;6(2):202–37. doi:10.1037/H0089739.

[36] Sandelowski M. Focus on research methods: whatever happened to qualitative description? Res Nurs Health. 2000;23(4):334–40. doi:10.1002/1098-240x(200008)23:4<334::aid-nur9>3.0.co;2-g. 10940958

[37] Soltani S, Killackey T, Birnie KA, Brennenstuhl S, Choiniere M, Pagé G, Dassieu L, Lacasse A, Lalloo C, Poulin P, et al. Impact of the COVID-19 pandemic on youth with chronic pain, parents, and siblings: a pan-Canadian study. Manuscript Submitted for Publication [European Journal of Pain]. 10.1002/ejp.2157 37435883

[38] Harris PA, Taylor R, Minor BL, Elliott V, Fernandez M, O’Neal L, McLeod L, Delacqua G, Delacqua F, Kirby J, et al. The REDCap consortium: building an international community of software platform partners. J Biomed Inform. 2019; 95:103208. doi:10.1016/j.jbi.2019.103208. 31078660 PMC7254481

[39] Song XJ, Xiong DL, Wang ZY, Yang D, Zhou L, Li RC. Pain management during the COVID-19 pandemic in China: lessons learned. Pain Med. 2020;21(7):1319–23. doi:10.1093/pm/pnaa143. 32321173 PMC7188156

[40] Killackey T, Baerg K, Dick B, Lamontagne C, Poolacherla R, Allen Finley G, Noel M, Birnie KA, Choinière M, Gabrielle Pagé M, et al. Experiences of pediatric pain professionals providing care during the COVID-19 pandemic: a qualitative study. Children (Basel). 2022;9(2). doi:10.3390/CHILDREN9020230. PMC8870259 35204950

[41] Patterson J. The role of family meanings in adaptation to chronic illness and disability. In: Turnbull A, Patterson J, Behr S, editors. Cognitive coping research and developmental disabilities. Baltimore: Brookes; 1993. p. 221–38.

[42] Lin M, Lo LY, Lui PY, Wong YK. The relationship between family resilience and family crisis: an empirical study of chinese families using family adjustment and adaptation response model with the family strength index. 2016;27(3):200–14. doi:10.1080/0897535320161199770.

[43] Braun V, Clarke V. Thematic analysis. In: APA handbook of research methods in psychology, Vol 2: research designs: quantitative, qualitative, neuropsychological, and biological. Washington (DC, US): American Psychological Association; 2012. p. 57–71. doi:10.1037/13620-004.

[44] Braun V, Clarke V. Reflecting on reflexive thematic analysis. Qual Res Sport Exerc Health. 2019;11(4):589–97. doi:10.1080/2159676X.2019.1628806.

[45] Dedoose Version 8.3.47. Web application for managing, analyzing, and presenting qualitative and mixed method research data; 2021. https://www.dedoose.com/

[46] Braun V, Clarke V. Using thematic analysis in psychology. Qual Res Psychol. 2006;3(2):77–101. doi:10.1191/1478088706qp063oa.

[47] Forgeron P, Higginson A, Truskoski C. Departure from pediatric care: transitioning of adolescents with chronic pain to adult care. Pain Manag Nurs. 2017;18(5):273–77. doi:10.1016/J.PMN.2017.05.001. 28778412

[48] Tham SW, Murray CB, Law EF, Slack KE, Palermo TM. The impact of the coronavirus disease 2019 pandemic on pain and psychological functioning in young adults with chronic pain. 2022. doi:10.1097/j.pain.0000000000002618. PMC9470785 35413028

[49] Neville A, Lund T, Soltani S, Jordan A, Stinson J, Killackey T, Birnie KA, Noel M. Pediatric chronic pain in the midst of the COVID-19 pandemic: lived experiences of youth and parents. J Pain. 2022;23(5):841–51. doi:10.1016/J.JPAIN.2021.11.012. 34915200 PMC8710941

[50] Hooten WM. Chronic pain and mental health disorders: shared neural mechanisms, epidemiology, and treatment. Mayo Clin Proc. 2016;91(7):955–70. doi:10.1016/J.MAYOCP.2016.04.029/ATTACHMENT/7B39E897-806F-4CA1-9F02-8742FCA5DC68/MMC1.MP4. 27344405

[51] Meulders A, Vlaeyen JWS, Evers AWM, Köke AJA, Smeets RJEM, van Zundert JHM, Verbunt JMCF, van Ryckeghem D. Chronic primary pain in the COVID-19 pandemic: how uncertainty and stress impact on functioning and suffering. Pain. 2022;163(4):604–09. doi:10.1097/J.PAIN.0000000000002428. 34382606

[52] Forgeron PA, King S, Stinson JN, McGrath PJ, MacDonald AJ, Chambers CT. Social functioning and peer relationships in children and adolescents with chronic pain: a systematic review. Pain Res Manag. 2010;15(1):27–41. doi:10.1155/2010/820407. 20195556 PMC2855293

[53] Lewandowski AS, Palermo TM, Stinson J, Handley S, Chambers CT. Systematic review of family functioning in families of children and adolescents with chronic pain. J Pain. 2010;11(11):1027–38. doi:10.1016/J.JPAIN.2010.04.005. 21055709 PMC2993004

[54] Palermo TM, Eccleston C. Parents of children and adolescents with chronic pain. Pain. 2009;146(1–2):15–17. doi:10.1016/J.PAIN.2009.05.009. 19482426 PMC2760649

[55] Datz H, Tumin D, Miller R, Smith TP, Bhalla T, Tobias JD. Pediatric chronic pain and caregiver burden in a national survey. Scand J Pain. 2019;19(1):109–16. doi:10.1515/SJPAIN-2018-0121/MACHINEREADABLECITATION/RIS. 30240360

[56] Eccleston C, Crombez G, Scotford A, Clinch J, Connell H. Adolescent chronic pain: patterns and predictors of emotional distress in adolescents with chronic pain and their parents. Pain. 2004;108(3):221–29. doi:10.1016/J.PAIN.2003.11.008. 15030941

[57] Palermo TM, Valrie CR, Karlson CW. Family and parent influences on pediatric chronic pain. Am Psychol. 2014;69(2):142–52. doi:10.1037/A0035216. 24547800 PMC4056332

[58] Yang XY, Gong RN, Sassine S, Morsa M, Tchogna AS, Drouin O, Chadi N, Jantchou P. Risk perception of COVID-19 infection and adherence to preventive measures among adolescents and young adults. Children. 2020;7(12):311. doi:10.3390/CHILDREN7120311. 33371272 PMC7766485

[59] Dkhar SA, Quansar R, Saleem SM, Khan SMS. Knowledge, attitude, and practices related to COVID-19 pandemic among social media users in J&K, India. Indian J Public Health. 2020;64(6):205. doi:10.4103/IJPH.IJPH_469_20. 32496256

[60] Clauw DJ, Häuser W, Cohen SP, Fitzcharles MA. Considering the potential for an increase in chronic pain after the COVID-19 pandemic. Pain. 2020;161(8):1694–97. doi:10.1097/J.PAIN.0000000000001950. 32701829 PMC7302093

[61] Quinlan-Colwell A, Schreier A. Pain management during the COVID-19 pandemic. Pain Manag Nurs. 2022;23(1):1–2. doi:10.1016/J.PMN.2021.12.001. 35093236 PMC8790054

[62] Jetha A, Tucker LB, Chen C, Gignac MAM. Impact of the COVID-19 pandemic on the employment of Canadian young adults with rheumatic disease: findings from a longitudinal survey. Arthritis Care Res (Hoboken). 2021;73(8):1146–52. doi:10.1002/ACR.24617. 33973377 PMC8212105

[63] Shanthanna H, Strand NH, Provenzano DA, Lobo CA, Eldabe S, Bhatia A, Wegener J, Curtis K, Cohen SP, Narouze S. Caring for patients with pain during the COVID-19 pandemic: consensus recommendations from an international expert panel. Anaesthesia. 2020;75(7):935–44. doi:10.1111/ANAE.15076. 32259288 PMC7262200

[64] Cascella M, Miceli L, Cutugno F, Di Lorenzo G, Morabito A, Oriente A, Massazza G, Magni A, Marinangeli F, Cuomo A. A delphi consensus approach for the management of chronic pain during and after the COVID-19 era. Int J Environ Res Public Health. 2021;18(24):13372. doi:10.3390/IJERPH182413372. 34948983 PMC8706033

[65] Harnik MA, Blättler L, Limacher A, Reisig F, Grosse Holtforth M, Streitberger K. Telemedicine for chronic pain treatment during the COVID-19 pandemic: do pain intensity and anxiousness correlate with patient acceptance? Pain Pract. 2021;21(8):934–42. doi:10.1111/PAPR.13071. 34463025 PMC8646884

[66] Hurley-Wallace AL, Nowotny E, Schoth DE, Liossi C. Online multidisciplinary interventions for paediatric chronic pain: a content analysis. Eur J Pain Suppl. 2021;25(10):2140–54. doi:10.1002/EJP.1827. 34155745

[67] Prime H, Wade M, Browne DT. Risk and resilience in family well-being during the COVID-19 pandemic. Am Psychol. 2020;75(5):631. doi:10.1037/AMP0000660. 32437181

[68] Dvorsky MR, Breaux R, Becker SP. Finding ordinary magic in extraordinary times: child and adolescent resilience during the COVID-19 pandemic. Eur Child Adolesc Psychiatry. 2020;30(11):1829–31. doi:10.1007/S00787-020-01583-8. 32613259 PMC7327857

[69] Bravo L, Killela MK, Reyes BL, Santos KMB, Torres V, Huang CC, Jacob E. Self-management, self-efficacy, and health-related quality of life in children with chronic illness and medical complexity. J Pediatr Health Care. 2020;34(4):304–14. doi:10.1016/J.PEDHC.2019.11.009. 32107073 PMC8381929

[70] Ghotbi T, Salami J, Kalteh EA, Ghelichi-Ghojogh M. Self-management of patients with chronic diseases during COVID19: a narrative review. J Prev Med Hyg. 2021;62(4):E814. doi:10.15167/2421-4248/JPMH2021.62.4.2132. 35603256 PMC9104668

[71] Kong H, Liu Y, Wu K, Cui S, Bai J, Fan X. Pain and self-management status among chinese patients with cancer during the COVID-19 pandemic. Pain Manag Nurs. 2022;23(1):26. doi:10.1016/J.PMN.2021.09.004. 34756521 PMC8487793

[72] Mun CJ, Campbell CM, McGill LS, Aaron RV. The early impact of COVID-19 on chronic pain: a cross-sectional investigation of a large online sample of individuals with chronic pain in the United States, April to May, 2020. Pain Med. 2021;22(2):470–80. doi:10.1093/PM/PNAA446. 33537764 PMC7901854

[73] Leonardi M, Horne AW, Vincent K, Sinclair J, Sherman KA, Ciccia D, Condous G, Johnson NP, Armour M. Self-management strategies to consider to combat endometriosis symptoms during the COVID-19 pandemic. Hum Reprod Open. 2020;2020(2). doi:10.1093/HROPEN/HOAA028. PMC7263080 32509977

[74] Cattelino E, Testa S, Calandri E, Fedi A, Gattino S, Graziano F, Rollero C, Begotti T. Self-efficacy, subjective well-being and positive coping in adolescents with regard to Covid-19 lockdown. Curr Psychol. 2021:1–12. doi:10.1007/S12144-021-01965-4/FIGURES/1. PMC8214713 34177208

[75] Damush TM, Kroenke K, Bair MJ, Wu J, Tu W, Krebs EE, Poleshuck E. Pain self-management training increases self-efficacy, self-management behaviours and pain and depression outcomes. Eur J Pain Suppl. 2016;20(7):1070–78. doi:10.1002/EJP.830. 26849410

[76] Gass K, Jenkins J, Dunn J. Are sibling relationships protective? A longitudinal study. J Child Psychol Psychiatry. 2007;48(2):167–75. doi:10.1111/j.1469-7610.2006.01699.x. 17300555

[77] Lee S, Dick BD, Jordan A, McMurtry CM. Psychological interventions for parents of youth with chronic pain: a scoping review. Clin J Pain. 2021;37(11):825–44. doi:10.1097/AJP.0000000000000977. 34475341

[78] Gayatri M, Irawaty DK. Family resilience during COVID-19 pandemic: a literature review. 2021;30(2):132–38. 10.1177/10664807211023875. PMC8980846 35399750

[79] Khanijahani A, Iezadi S, Gholipour K, Azami-Aghdash S, Naghibi D. A systematic review of racial/ethnic and socioeconomic disparities in COVID-19. Int J Equity Health. 2021;20(1):1–30. doi:10.1186/S12939-021-01582-4/TABLES/2. 34819081 PMC8611382

[80] Woollam M, Angarita-Rivera P, Siegel AP, Wu K, Jelfs B, Ma X, Paul A, Englert P, Varga M. Socio-economic disparities and COVID-19 in the USA. J Phys Complex. 2021;2(3):035017. doi:10.1088/2632-072X/AC0FC7.

